# Postmarketing Reevaluation of Chinese Traditional Therapy Kangbingdu Oral Liquid in the Treatment of the Common Cold

**DOI:** 10.1155/2022/9968171

**Published:** 2022-09-01

**Authors:** Hongjiao Li, Yanke Ai, Tianyi Zhao, Di Zhang, Xiaoying Lv, Shaoyan Jia, Zehuai Wen, Guoxin Li, Hongyu Wang, Feng Gao, Shaohong Li, Zhishan Ge, Yuning Qin, Zhenbiao Wang, Liyun He

**Affiliations:** ^1^Institute of Basic Research in Clinical Medicine, China Academy of Chinese Medical Sciences, Beijing, China; ^2^Hospital of Beihang University, Beijing, China; ^3^Guangdong Provincial Hospital of Chinese Medicine, Guangzhou, China; ^4^Second Teaching Hospital of Liaoning University of Traditional Chinese Medicine, Shenyang, China; ^5^Hospital of Renmin University of China, Beijing, China; ^6^WangJing Hospital of China Academy of Chinese Medical Sciences, Beijing, China; ^7^Beijing ShiJiTan Hospital, Capital Medical University, Beijing, China

## Abstract

**Background:**

Observational studies from China suggest that Kangbingdu oral liquid (KBD) may be effective in treating the common cold.

**Objective:**

Reevaluation of efficacy and safety of Kangbingdu oral liquid after marketing and expanding population.

**Design:**

Prospective, Pragmatic randomized controlled trial (Chictr.org.cn registration number: chiCTR-TRC-12002399). *Setting*. Eleven hospitals from 3 provinces in China. Patients were recruited through 11 centers, including 7 teaching hospitals, 2 University health services, one military clinic, and one community hospital. *Patients*. 2647 persons aged 18 to 75 years with Common cold. *Intervention*. Patients were randomly allocated to 2 groups: the treatment group Kangbingdu oral liquid (composed of 9 Chinese herbal medicines and honey) and the placebo group were divided into a standard-dose group of 10 ml every time, a middle dose group of 20 ml every time, high dose group of 30 ml every time, 3 times daily. Interventions and control were given for 5 days. *Measurements*. The primary outcome is the mean amount of total scores measured by the 11-primary symptoms: to observe the change of main symptoms from severe to disappear and to calculate and compare the mean amount of total scores after the periods of observation. Secondary outcomes are the disappearance rate of each symptom and the median time of body temperature returned to normal.

**Results:**

On day 5, the Kangbingdu liquid group had significant reductions in the mean amount of total scores measured by the 11-primary symptoms (7.39 [95% CI 7.26 to 7.51] compared to the placebo group (6.43 [95%: CI 6.24 to 6.62]). The Kangbingdu liquid can improve the remission rate of accompanying symptoms on day 5 including aversion to wind, aversion to cold, fever, cough, stuffy, runny nose, sore throat, muscular aches, headache, fatigue, and sweat (*P* < 0.0001). Significant reductions in time of body temperature to return to normal in the Kangbingdu liquid group (P50, 48.33 [95% CI 46.00 to 52.50] compared with the control group (P50, 64.59 [95% CI 51.08 to 70.50] (*P*=0.0022). 13 (0.7%) participants in the Kangbingdu liquid group and 1(0.2%) participants in the placebo group (*P* > 0.05) had treatment-related AEs, which mainly include diarrhea and dyspepsia in the Kangbingdu liquid group and constipation in the placebo group.

**Conclusion:**

The study's conclusion in this paper was based on the placebo, Kangbingdu oral liquid two groups which clinically diagnosed the common cold and flu. (1) Kangbingdu oral liquid can effectively improve the comprehensive clinical symptoms of common adult cold, also improved main symptoms, including sore throat, muscle aches, headache, and so on. (2) Kangbingdu oral liquid effectively shortens the time of body temperature to return to normal.

## 1. Introduction

A common cold is associated with significant morbidity and economic consequences. The common cold is the most common respiratory illness and medical condition [1]. Rhinoviruses mainly cause colds and other pathogens, such as respiratory syncytial virus, adenovirus, and coronavirus, are also common pathogens [2, 3]. In the United States, adults catch about 4–6 colds a year, and children catch about 6–8 colds a year. On average, 8.7 hours of working time will be wasted daily due to the cold, including 5.9 hours of on-the-job loss and 2.8 hours of absenteeism [4,5]. There are about 110 million general visits and 6 million emergency visits due to colds yearly, causing an economic burden of about 40 billion US dollars [6]^.^ Colds with a longer duration, worsening, and frequency will prolong the presence of eosinophilic neutrophils in the nose, making asthma challenging to control and exacerbating COPD severity [7–9].

Kangbingdu (KBD) oral liquid, a classic traditional Chinese medicinal formula that is revised based on the traditional Chinese medicine (TCM) formulations of “BaiHuTang” and “QingWenBaiDuYin,” is widely used for the clinical treatment of influenza and common cold [10,11].

## 2. Methods

### 2.1. Study Design

We conducted a prospective, randomized, double-blind, placebo-controlled trial at 11 medical sites in three provinces in China. The plan was included in the sample of 2647 cases in this test, using the stratified-block randomization method, with fever and no fever. Each subgroup met 100 samples, and the central randomization system was used to screen subjects, randomize subjects, and dispense drugs. The institutional review board of the Institute of Basic Research in Clinical Medicine (IBRCM) reviewed and approved the protocol and consent forms before the start of the study. All participants signed written informed consent forms before enrollment.

### 2.2. Patient Enrollment

A Consensus Recommendation from an Expert Panel for Primary Care Clinicians [12]. They usually aversion to wind, fever, cough, stuffy nose, runny nose, sore throat, muscle aches, headaches, tiredness, sweat, and so on. Patients who fulfilled all of the following criteria were included: patients in the studyaged 18 to 75 years and had a clinical diagnosis of influenza or common cold within 48 hours of the onset of symptoms of common cold or influenza; written informed consent was obtained; and without other prevention and control of cold drugs, including antibiotics, regularly in the 48 hours before inclusion.

Exclusion criteria were as follows:(1) Patients with pneumonia, bronchitis, otitis media, pharyngeal isthmus, viral myocarditis, acute nephritis, rheumatic joint disease, and other diseases.(2) Patients had hepatic and renal insufficiency; (3) Patients with allergies to clear antivirals in KBD components (plate blue root, gypsum, lugan, dihuang, turmeric, cicadas, rock calamus, patchouli, and forsythia). (4) Patients were pregnant or lactating women; had intellectual or behavioral dysfunction and were unable to cooperate with the completion of the clinical observer. (5) During a month, patients had taken part in other clinical studies. (6) Blood routine detection indicated a white blood cell count above 12^*∗*^10^9^/L; had antibiotics or other anticold medicines continuously for 48 hours; had other lesions or feelings that reduced the possibility of complicating.

### 2.3. Drug Administration

KBD and placebo that we used in our study were manufactured by Guangzhou Xiangxue Pharmaceutical Co., Ltd. KBD is composed of 9 herbs Radix isatidis (Banlangen; the radix of *Isatis indigotica* Fortune ex Lindl. of family Brassicaceae), Rhizoma phragmitis (Lugen; the rhizome of *Phragmites communis* Trin of family Poaceae), Radix Rehmanniae (Dihuang; the radix of *Rehmannia glutinosa* (Gaertn.) DC of family Scrophulariaceae), Radix Curcumae (Yujin; the radix of *Curcuma wenyujin* Y. H. Chen et al., C. Ling, a synonim of the accepted name *Curcuma aromatica* Salisb. According to theplantlist.org, of family Zingiberaceae), Rhizoma Anemarrhenae (Zhimu; the rhizome of *Anemarrhena asphodeloides*. Bunge of family Asparagaceae), Rhizoma acori tatarinowii (Shichangpu; the rhizome of *Acorus tatarinowii* Schott, a synonym of the accepted name Acorus calamus L, of family Acoraceae), Herba pogostemonis (Guanghuoxiang; the caulis of *Pogostemon cablin* (Blanco) Benth. of family Lamiaceae), Fructus Forsythiae (Lianqiao; the fructus of *Forsythia suspensa* (Thunb.) Vahl. of family Oleaceae), and Gypsum fibrosum (Shigao; one mineral with hydro calcium sulfate fibriform crystallized polymeric) in a dry weight of 129 g, 61 g, 32 g, 25 g, 25 g, 25 g, 29 g, 46 g and 57 g, respectively. The criteria for the quality of the herbs we used were by the 2005 Chinese pharmacopeia. And placebo keeps the color and taste the same as KBD.

Laboratory personnel was blinded to the identity of the groups. At each study site, a trained technician distributes drugs to subjects. After agreeing to participate, signing the informed consent form, and completing the baseline visit, all patients were randomly assigned to treatment groups or the control group by using random-number tables with a block size of 1 (SPSS software, version 13.0 [SPSS, Chicago, Illinois]. Randomization was stratified by the 11 study centers located in Beijing, Shenyang, and Guangzhou. The centers were selected to ensure the geographic spread and representation of common cold and influenza epidemic areas in mainland China. Participants were given placebos or KBD daily for 5 days, and participants were allowed to use ibuprofen if their body temperature was greater than 38°C. Each site had a coordinator who was assigned to follow-up on the participants' treatment and symptoms by telephoning.

### 2.4. Primary Outcomes

For the study's primary symptoms, we established an efficacy evaluation scale. At the study design stage, we convened an expert consultation session with clinical and methodological specialists on the efficacy assessment indexes of oral antiviral treatments to rationalize the selection of efficacy evaluation indexes. Considering the contrasts between TCM and contemporary medicine's perspectives on the diagnosis and treatment of colds, symptoms such as aversion to wind, cold, and sweat are TCM diagnosis-specific symptoms. These symptoms are not included in the Western medicine consensus [12] or guidelines [13] on colds. As a result, we devised our own scale based on the most prevalent symptoms of common cold and complemented the primary symptoms in prior study reports from doctors and patients on the symptoms of the common cold. We finally formed the efficacy evaluation scale, which included 11 main symptoms of common cold patients in China, namely, aversion to wind, aversion to cold, fever, cough, stuffy nose, runny nose, sore throat, muscle aches, headache, fatigue, and sweat, when combined with the results of literature analysis.

Given that it is common for physicians and patients to describe the degree of disease as “mild, moderate, or severe” in clinical practice, we adopted a 4-point scale. We have adopted a 4-point scale to assess the severity of symptoms, described as follows: (1) None : no symptoms of the condition. (2)Slightly: the symptom is present but occurs infrequently or very mildly. (3) More clearly: the symptom is present but not severe, between mild and severe, and generally tolerable. (4) Severe: the frequency or/and intensity of the symptom is very severe and significantly affects work and life. None, slightly, more clearly, and severe were assigned according to 0, 1, 2, and 3 points, with a maximum total score of 44 points for the 11 major symptoms.

### 2.5. Assessment Process

Patients and researchers conducted the evaluations. Before the study begins, all 11 centers' researchers must complete standard operating procedures (SOP) training, and records must be kept in accordance with SOP. Patients in this study were given patient-administered diaries, and coordinators instructed them on how to fill the diaries according to the trial's protocols. A case report form(CRF) must be filled out by research.

The treatment period from the first day to the fifth day, as well as the interview, were conducted over the phone by the coordinator from the second to the fourth day, with the first and last days requiring face-to-face visits, with the last visit being automatically postponed to Monday in the event of a weekend. Patients were given test drugs on the first visit, and researchers should remind them to return all of the leftover test medications and packing boxes on the last visit. Clearly document the amount of distributing medicines that patients used during the experiment if drug recovery was challenging.

### 2.6. Statistical Analysis

The sample size is not based entirely on statistical considerations. The clinical trial included 2800 cases and ensured that 2600 cases were effective. The trial will be over once the situation is satisfying.

All analyses were based on the intention-to-treat (ITT) principle. For the primary outcome, we used a mixed-effect model with baseline value as a covariate, therapy, and site as fixed effects to examine the change in total symptom score from baseline on day 5. For missing data on the primary outcome, we employed the last observation carried forward (LOCF) approach.

We used the Chi-square test or Fisher exact test to compare the disappearance rate of single symptoms at baseline and the log-rank test to compare the time it took for body temperature to return to normal for fever cases at baseline. Secondary outcomes were performed in the observed cases without imputation of missing data.

All analyses were performed with SAS version 9.4 (SAS Institute, Cary, NC, USA), with a 2-sided *P* value of less than 0.05 considered significant. No adjustment was made for multiple comparisons and interim analysis.

## 3. Results

**Figure 1:**
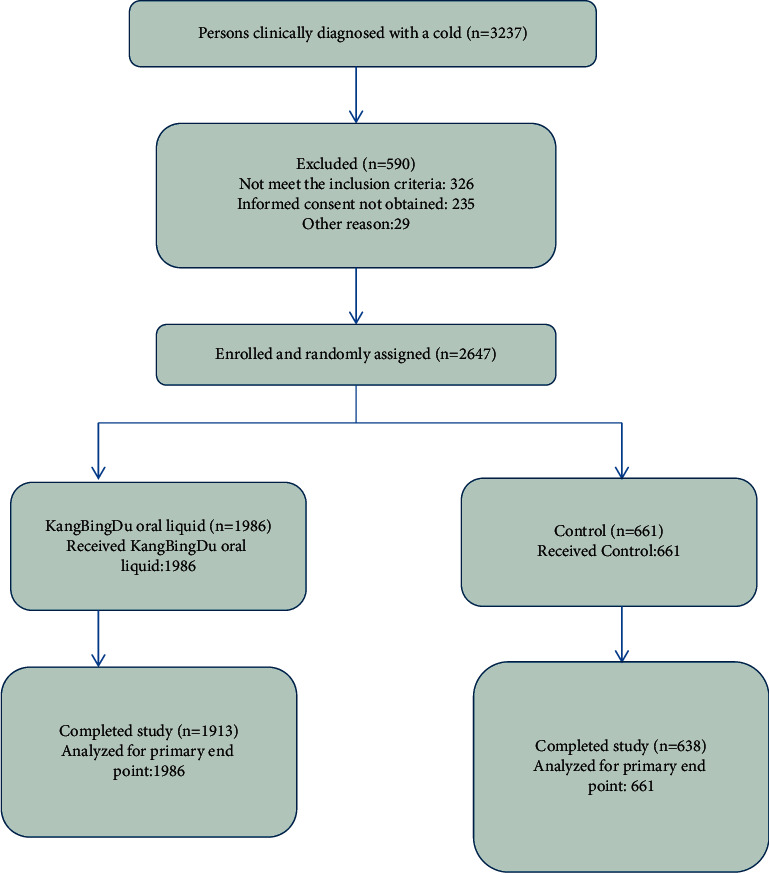
Study flow diagram.

Between December 12th, 2012, and December 28th, 2015, we screened 3237 participants and randomly assigned 2647 participants to either the Kangbingdu liquid group (*n* = 1986) or the placebo (*n* = 661) group. Among the randomized participants, 1913 (96.3%) in the group and 638 (96.4%) in the placebo group completed the study (Figure 1). Baseline characteristics of the participants were similar between groups (Table 1).

 (Figure 1) (Table 1)

For the primary outcome, the mean number of total scores measured by the 11-primary symptoms was 8.5 (95%CI, 8.3 to 8.7) at baseline and 1.1 (95%CI, 1.0 to 1.2) on day 5 in the Kangbingdu liquid group; the mean number of total scores measured by the 11-symptoms was 8.4 (95%CI, 8.1 to 8.7) at baseline and 2.0 (95%CI, 1.8 to 2.3) at day 5 in the placebo group. The reduction in total scores was greater in the Kangbingdu liquid group (7.39, 95% CI, 7.26 to 7.51) than in the placebo group (6.43, 95% CI, 6.24 to 6.62), with a mean difference of 0.96 (95% CI, 0.75 to 1.16; *P* < 0.0001) (Table 2). When compared to the placebo group, the Kangbingdu liquid group showed a higher rate of symptom disappearance (Table 2). The median time for body temperature returned to normal was 48.33 (46.00 to 52.50) hours in the Kangbingdu liquid group, compared with 64.59 (51.08 to 70.50) hours in the placebo group (*P*=0.0022) (Table 2).

During the trial, 13 (0.7%) participants in the Kangbingdu liquid group and 1 (0.2%) participant in the placebo group (*P* > 0.05) had treatment-related AEs, which mainly included diarrhea and dyspepsia in the Kangbingdu liquid group and constipation in the placebo group. Two participants, all from the Kangbingdu liquid group, withdrew from the study because of adverse events (all for no-treatment-related stomachaches and dizziness) (Table 3).

## 4. Discussion

To our knowledge, this trial is one of the few prospective, randomized, double-blind, placebo-controlled trials investigating the efficacy and safety of TCM clinical treatment for influenza and the common cold in China. We found that the improvement in common cold-related symptoms [12]was significantly more rapid with KBD. Compared with placebo, KBD can decrease the mean total score of 11-main symptoms.

The common cold mainly affects the upper respiratory tract and is typically characterized by nasopharyngeal catarrhal symptoms. Sore throat, stuffy nose, runny nose, cough, and fatigue usually peak in 1–3 days and last 7–10 days but can last for several weeks [14,15]. Coughs might linger longer than other cold symptoms, and they can cause sleep deprivation, myalgia, urine incontinence (particularly in women), and anxiety [16,17]. According to the survey, 52 percent of cold patients say their symptoms have a substantial influence on their everyday lives, and 93 percent are unable to work properly owing to sleeping problems caused by a cough or stuffy nose [18].

Treatment focuses on symptom improvement. While nonsteroidal anti-inflammatory drugs do not reduce the total symptom score of cold patients, they also increase the score of some symptoms, such as sneezing [19]. The nasal constrictor can relieve congestion, but not cough [20]. It also increases the risk of rhinitis [21]. In the following two surveys on cold medicine choices, Chinese people were more likely to take proprietary traditional Chinese medicine or a combination of drugs to treat common colds.

Investigation and analysis of medication for common colds among students in school revealed that 97.12% of medical students and 85.96% of nonmedical students chose the self-purchase medicine types containing “proprietary Chinese medicine” or “traditional Chinese medicine decoction,” indicating that the use of Traditional Chinese medicine in the treatment of colds is relatively popular among students in our school, and most students recognize the benefits. [22]. Students knew about medications mostly from pharmacists in drugstores, doctors, and commercials. Traditional Chinese Medicine decoctions and Chinese and Western medicine mixtures were their favored drug types [23].

In our trial, the Kangbingdu Liquid group(7.39, 95% CI, 7.26 to 7.51) signiﬁcantly performed better than the placebo group (6.43, 95% CI, 6.24 to 6.62) in terms of total symptom score reduction on day five compared to baseline with a mean difference of 0.96 (95% CI, 0.75 to 1.16; P.0001).

The mechanism of TCM in the treatment of influenza is complex. A pharmacodynamic study of Kangbingdu oral liquid shows that Kangbingdu oral liquid has sound therapeutic and preventive effects on influenza A (H1N1) virus infection in vivo and in vitro [24]. Administration of Chinese herbs may have beneficial immunomodulatory effects for the rapid recovery of viral infections.

There were 14 adverse events in our trial (13 in the Kangbingdu liquid group and one in the placebo group). The adverse events observed in the KBD group were also reported in other KBD studies or reports [25,26], and none of the adverse events affected the progress of the study.

There are certain limitations to our research. Our study participants were mostly young and had been clinically diagnosed with the common cold. They went to the hospital to get relief from their symptoms and prevent aggravating their symptoms, which could lead to the development of other ailments. Following that, we intend to conduct real-world research to determine the appropriate population of KBD and, based on that, utilize positive control medications to evaluate the antiviral effect of KBD in clinical trials.

## 5. Conclusions

Through this trial, Kangbingdu oral liquid effectively improved the comprehensive clinical symptoms of an adult common cold, also improving the main symptoms including sore throat, muscle aches, headache, and so on. Kangbingdu oral liquid effectively shortens the time it takes for the body temperature to return to normal.

## Figures and Tables

**Table 1:** Demographic characteristics and baseline data.

Characteristics	Kangbingdu liquid (*n* = 1986)	Placebo (*n* = 661)	*P* value
Age, mean (SD)	36.1 (14.90)	36.1 (14.81)	0.7105

Sex Female Male	893 (45.12%) 1086 (54.88%)	303 (45.84%) 358 (54.16%)	0.9106

Race			0.2337
Han	1924 (96.88%)	631 (95.46%)	
Others	62 (3.12%)	30 (4.54%)	

Marriage			0.7909
Yes	1081 (54.43%)	361 (54.61%)	
No	905 (45.57%)	300 (45.39%)	

Clinic diagnosis			0.1080
Common cold	1857 (93.50%)	616 (93.19%)	
Flu	129 (6.50%)	45 (6.81%)	
Highest body temperature	37.1 ± 0.73	36.9 ± 0.70	0.5693

Fever			
No	1274 (64.15%)	418 (63.24%)	
Yes	712 (35.85%)	243 (36.76%)	

Time from cold to visit the doctor			0.9682
≤ 24h	1283 (64.60%)	393 (59.46%)	
> 24h且 ≤ 48h	703 (35.40%)	268 (40.54%)	

Influenza exposure in the week prior			0.0639
No	1808 (91.04%)	593 (89.71%)	
Yes	178 (8.96%)	68 (10.29%)	

Influenza vaccinations			0.5497
No	1698 (85.50%)	561 (84.87%)	
Yes	117 (5.89%)	42 (6.35%)	
Total score, mean (SD)	8.5 (4.59)	8.4 (4.21)	

Aversion to wind			0.9626
None	1156 (58.21%)	380 (57.49%)	
Slightly	574 (28.90%)	205 (31.01%)	
More clearly	213 (10.73%)	60 (9.08%)	
Severe	43 (2.17%)	16 (2.42%)	

Aversion to cold			0.9992
None	1074 (54.08%)	357 (54.01%)	
Slightly	602 (30.31%)	202 (30.56%)	
More clearly	261 (13.14%)	85 (12.86%)	
Severe	49 (2.47%)	17 (2.57%)	

Fever			0.9433
None	1181 (59.47%)	394 (59.61%)	
Slightly	380 (19.13%)	129 (19.52%)	
More clearly	394 (19.84%)	123 (18.61%)	
Severe	31 (1.56%)	15 (2.27%)	

Cough			0.1526
None	823 (41.44%)	251 (37.97%)	
Slightly	704 (35.45%)	246 (37.22%)	
More clearly	380 (19.13%)	141 (21.33%)	
Severe	79 (3.98%)	23 (3.48%)	

Stuffy nose None slightly More clearly Severe	668 (33.64%) 728 (36.66%) 520 (26.18%) 70 (3.52%)	246 (37.22%) 232 (35.10%) 164 (24.81%) 19 (2.87%)	0.1044

Runny nose			0.5931
None	594 (29.91%)	188 (28.44%)	
Slightly	718 (36.15%)	245 (37.07%)	
More clearly	571 (28.75%)	194 (29.35%)	
Severe	103 (5.19%)	34 (5.14%)	

Sore throat			0.5173
None	556 (28.00%)	186 (28.14%)	
Slightly	601 (30.26%)	215 (32.53%)	
More clearly	719 (36.20%)	222 (33.59%)	
Severe	110 (5.54%)	38 (5.75%)	

Muscle aches			0.4802
None	1076 (54.18%)	371 (56.13%)	
Slightly	570 (28.70%)	178 (26.93%)	
More clearly	303 (15.26%)	98 (14.83%)	
Severe	37 (1.86%)	14 (2.12%)	

Headache			0.4222
None	957 (48.19%)	323 (48.87%)	
Slightly	631 (31.77%)	221 (33.43%)	
More clearly	356 (17.93%)	110 (16.64%)	
Severe	42 (2.11%)	7 (1.06%)	

Fatigue			0.5125
None	826 (41.59%)	267 (40.39%)	
Slightly	787 (39.63%)	263 (39.79%)	
More clearly	333 (16.77%)	117 (17.70%)	
Severe	40 (2.01%)	14 (2.12%)	

Sweat			0.4521
None	1429 (71.95%)	485 (73.37%)	
Slightly	451 (22.71%)	145 (21.94%)	
More clearly	90 (4.53%)	26 (3.93%)	
Severe	16 (0.81%)	5 (0.76%)	

Total scores mean(95%CI)	8.50 (8.30 to 8.70)	8.42 (8.10 to 8.74)	0.6777

**Table 2:** Efficacy data.

Variable	Kangbingdu liquid	Placebo	Difference (95%CI)	*P* value
Primary outcome	*n* = 1986	*n* = 661		
Total- scores	8.5 (8.3 to 8.7)	8.4 (8.1 to 8.7)		
Total scores on day 5, mean(95%CI)	1.10 (1.00 to 1.20)	2.05 (1.82 to 2.28)		
Change on day 5, adjusted mean(95%CI)	7.39 (7.26 to 7.51)	6.43 (6.24 to 6.62)	0.96 (0.75 to 1.16)	<0.0001

Secondary outcomes				

Aversion to wind	n = 830 (%)	*n* = 281 (%)		
Symptom resolution rate on day 2	275 (33.13)	63 (22.42)	−10.71 (−16.55 to -−4.88)	0.0007
Symptom resolution rate on day 3	500 (60.24)	151 (53.74)	−6.5 (−13.22 to 0.21)	0.0557
Symptom resolution rate on day 4	672 (80.96)	204 (72.60)	−8.37 (-14.23 to −2.51)	0.0030
Symptom resolution rate on day 5	754 (90.84)	228 (81.14)	−9.7 (−14.68 to −4.73)	< 0.0001

Aversion to cold	*n* *=* 912 (%)	*n* = 304 (%)		
Symptom resolution rate on day 2	353 (38.71)	74 (24.34)	−14.36 (−20.13 to −8.6)	< 0.0001
Symptom resolution rate on day 3	606 (66.45)	156 (51.32)	−15.13 (−21.53 to −8.73)	< 0.0001
Symptom resolution rate on day 4	758 (83.11)	219 (72.04)	−11.07 (−16.67 to −5.47)	< 0.0001
Symptom resolution rate on day 5	843 (92.43)	258 (84.87)	−7.57 (−11.94 to −3.19)	< 0.0001

Fever	*n* = 805 (%)	*n* = 267 (%)		
Symptom resolution rate on day 2	321 (39.88)	63 (23.60)	−16.28 (−22.39 to −10.17)	< 0.0001
Symptom resolution rate on day 3	563 (69.94)	137 (51.31)	−18.63 (-25.41 to −11.85)	< 0.0001
Symptom resolution rate on day 4	700 (86.96)	205 (76.78)	−10.18 (−15.75 to −4.6)	< 0.0001
Symptom resolution rate on day 5	766 (95.16)	230 (86.14)	−9.01 (−13.41 to −4.61)	< 0.0001

Cough	*n* = 1163 (%)	*n* = 410 (%)		
Symptom resolution rate on day 2	225 (19.35)	49 (11.95)	−7.4 (−11.27 to −3.52)	0.0007
Symptom resolution rate on day 3	425 (36.54)	102 (24.88)	−11.67 (−16.68 to −6.65)	< 0.0001
Symptom resolution rate on day 4	614 (52.79)	168 (40.98)	−11.82 (−17.38 to −6.26)	< 0.0001
Symptom resolution rate on day 5	833 (71.63)	229 (55.85)	−15.77 (−21.23 to −10.31)	< 0.0001

Stuffy	*n* = 1318 (%)	*n* = 415 (%)		
Symptom resolution rate on day 2	288 (21.85)	47 (11.33)	−10.53 (−14.3 to −6.75)	< 0.0001
Symptom resolution rate on day 3	591 (44.84)	139 (33.49)	−11.35 (−16.62 to −6.07)	< 0.0001
Symptom resolution rate on day 4	876 (66.46)	228 (54.94)	−11.52 (−16.95 to −6.1)	< 0.0001
Symptom resolution rate on day 5	1133 (85.96)	295 (71.08)	−14.88 (−19.63 to −10.13)	< 0.0001

Runny nose	*n* = 1392 (%)	*n* = 473 (%)		
Symptom resolution rate on day 2	317 (22.77)	62 (13.11)	−9.67 (−13.42 to −5.91)	< 0.0001
Symptom resolution rate on day 3	639 (45.91)	161 (34.04)	−11.87 (−16.88 to −6.86)	< 0.0001
Symptom resolution rate on day 4	927 (66.59)	244 (51.59)	−15.01 (−20.15 to −9.87)	< 0.0001
Symptom resolution rate on day 5	1150 (82.61)	326 (68.92)	−13.69 (−18.31 to −9.07)	< 0.0001

Sore throat	*n* = 1430 (%)	*n* = 475 (%)		
Symptom resolution rate on day 2	301 (21.05)	64 (13.47)	−7.58 (−11.3 to −3.85)	0.0003
Symptom resolution rate on day 3	613 (42.87)	152 (32.00)	−10.87 (−15.78 to −5.95)	< 0.0001
Symptom resolution rate on day 4	928 (64.90)	252 (53.05)	−11.84 (−16.97 to −6.72)	< 0.0001
Symptom resolution rate on day 5	1183 (82.73)	330 (69.47)	−13.25 (−17.84 to −8.67)	< 0.0001

Muscular soreness	*n* = 910 (%)	*n* = 290 (%)		
Symptom resolution rate on day 2	356 (39.12)	66 (22.76)	−16.36 (−22.14 to −10.59)	< 0.0001
Symptom resolution rate on day 3	579 (63.63)	148 (51.03)	−12.59 (−19.14 to −6.04)	0.0001
Symptom resolution rate on day 4	757 (83.19)	211 (72.76)	−10.43 (−16.1 to −4.76)	< 0.0001
Symptom resolution rate on day 5	834 (91.65)	237 (81.72)	−9.92 (−14.72 to −5.13)	< 0.0001

Headache	*n* = 1029 (%)	*n* = 338 (%)		
Symptom resolution rate on day 2	427 (41.50)	94 (27.81)	−13.69 (−19.33 to −8.04)	< 0.0001
Symptom resolution rate on day 3	675 (65.60)	204 (60.36)	−5.24 (−11.21 to 0.73)	0.0809
Symptom resolution rate on day 4	847 (82.31)	252 (74.56)	−7.76 (−12.95 to −2.56)	0.0018
Symptom resolution rate on day 5	947 (92.03)	277 (81.95)	−10.08 (−14.5 to −5.66)	< 0.0001

Fatigue	*n* = 1160 (%)	*n* = 394 (%)		
Symptom resolution rate on day 2	395 (34.05)	79 (20.05)	−14 (−18.8 to −9.2)	< 0.0001
Symptom resolution rate on day 3	702 (60.52)	173 (43.91)	−16.61 (−22.26 to −10.96)	< 0.0001
Symptom resolution rate on day 4	874 (75.34)	259 (65.74)	−9.61 (−14.91 to −4.31)	0.0002
Symptom resolution rate on day 5	1019 (87.84)	301 (76.40)	−11.45 (−16.04 to −6.85)	< 0.0001

Sweat	*n* = 557 (%)	*n* = 176 (%)		
Symptom resolution rate on day 2	255 (45.78)	61 (34.66)	−11.12 (−19.28 to −2.96)	0.0094
Symptom resolution rate on day 3	404 (72.53)	102 (57.95)	−14.58 (−22.76 to −6.4)	0.0003
Symptom resolution rate on day 4	482 (86.54)	128 (72.73)	−13.81 (−20.97 to −6.64)	< 0.0001
Symptom resolution rate on day 5	503 (90.31)	141 (80.11)	−10.19 (−16.58 to −3.8)	0.0003

Time for body temperature to return to normal	*n* = 579	*n* = 196		
P25 (95%CI)	28.00 (25.37 to 31.50)	35.34 (26.00 to 43.17)		0.0022
P50 (95%CI)	48.33 (46.00 to 52.50)	64.59 (51.08 to 70.50)		

**Table 3:** Adverse events (AE).

	Kangbingdu Liquid (n = 1986)	Placebo (*n* = 661)			Total (*n* = 2647)	
	Number (%)	Cases	Number (%)	Cases	Number (%)	Cases
Total AE	24 (1.2)	25	3 (0.5)	3	27 (1.0)	28
AE is associated with the study of drug	13 (0.7)	13	1 (0.2)	1	14 (0.5)	14
AE leading to withdrawal	2 (0.1)	2	0 (0)	0	2 (0.1)	2

## Data Availability

The data used are available from the corresponding author upon request.
